# Reduced Chronic Obstructive Pulmonary Disease–Related Utilization of Health Care Services and Increased Social Activities by Patients Offered a 24/7 Accessible Telehealth Service Based on the Epital Care Model: Pragmatic Modified Stepped Wedge Randomized Controlled Trial

**DOI:** 10.2196/65300

**Published:** 2025-10-22

**Authors:** Klaus Phanareth, Gustav Thomsen Purreskov, Emil Fuhr Nielsen, August Toft Bentsen, Lone Schou, Stanton Newman, Lars Kayser

**Affiliations:** 1 Response and Coordination Center ECM Clinic Ltd. Copenhagen Denmark; 2 Department of Public Health University of Copenhagen Department of Public Health, University of Copenhagen Copenhagen Denmark; 3 School of Health & Psychological Sciences City St George's, University of London London United Kingdom; 4 Section of Health Services Research Department of Public Health, University of Copenhagen Copenhagen Denmark

**Keywords:** chronic obstructive pulmonary disease, exacerbation, telehealth, relapse prevention, Epital Care Model, patient-reported outcome measures, disease management, organization and administration

## Abstract

**Background:**

An escalating prevalence of older adults living longer with chronic conditions challenges the health care workforce. Innovative web-based services, such as those based on the Epital Care Model (ECM), may help address these challenges, though their effects remain undocumented.

**Objective:**

The objectives of this study are to investigate whether telehealth services provided by an ECM response and coordination center, complementary to usual care provided by general practitioners (GPs), affect the participants’ mental well-being and use of health care services as primary outcomes, and social activities and mobility as a secondary outcome.

**Methods:**

Using a pragmatic, modified, stepped wedged, nonblinded design, 184 people living with chronic obstructive pulmonary disease, diagnosed in accordance with the GOLD (Global Initiative for Chronic Obstructive Lung Disease) guidelines, were, for logistic and resource reasons, randomized over a period of 10 months, within blocks of up to ten participants and at four geographically distinct locations, into either an ECM-based complementary telehealth service (ECTHS), manned by certified nonprofessional staff, or usual care provided by GPs and other health care services. All baseline parameters were collected in person, whereas all follow-up data were based on data from the participants’ health records and telephone interviews at 8 months (T1) and 12 months (T2). Mental well-being was assessed by the World Health Organization-5 Well-Being Index (WHO-5), using an independent 2-sample *t* test for comparison between groups and a dependent 2-sample *t* test for comparison between T0 and T1 and T2, respectively, within the two groups. Health care service usage and participants’ social activity and mobility were assessed and compared using Poisson regression.

**Results:**

In an intention-to-treat analysis, there were no differences in WHO-5 score within or between groups, whereas a difference was found at T2 in a per protocol analysis (mean 68.68, SD 17.40 in ECTHS vs 59.70, SD 19.51 in usual care; *P*=.01). Estimates of the Poisson regression were for chronic obstructive pulmonary disease related contacts to health care services at T2 for hospital admissions (0.51; *P*=.04), out-of-office services (0.49; *P*=.09), outpatient clinics (0.49; *P*=.02), and visits at GP (0.25; *P*<.001), demonstrating a reduction in usage between 49% and 75% after adjustment for age, GOLD risk score, risk time and comorbidities. In addition, at both T1 and T2, there was an increase in participation in cultural events and travel activities abroad, with T2 estimates of 1.73 (*P*<.001) and 2.50 (*P*=.002), respectively, demonstrating an increase of 73% and 150%, respectively.

**Conclusions:**

These results contribute to a new perspective on how health care services can be organized to reduce health care usage and increase social activity and mobility based on an ECTHS manned with nonprofessional certified staff.

**Trial Registration:**

ClinicalTrials.gov NCT06988566, https://clinicaltrials.gov/study/NCT06988566 (retrospectively registered)

## Introduction

Western health care systems are under huge pressure with an aging population, health care provider burnout, workforce shortages, supply chain disruptions, equipment shortages, lack of hospital beds, and outdated facilities [1]. Of these, the aging population and its impact is by far one of the largest challenges for health care systems in high- and middle-income countries. The global population of older adults, aged 80 years or older, is expected to triple between 2020 and 2050 to reach 426 million by 2050 [2]. Due largely to aging and its association with increased chronic disease, pressures on health care systems are substantial. In addition, most health care systems are experiencing a shortage of health care professionals. These factors have led to a search for innovative and effective solutions.

In this context, digital health services and new technologies, including telehealth, may, if appropriately implemented, be of advantage to older people with long-term health conditions (LTHC) and may facilitate easier and more efficient access to health services [3].

One of the largest challenges of LTHC is chronic obstructive pulmonary disease (COPD), which is the third leading cause of death worldwide, causing 3.23 million deaths in 2019 [4,5]. COPD is characterized by a gradual deterioration with progressive loss of pulmonary function over time, with intermittent episodes of exacerbations [4,6].

The treatment of COPD exacerbations (ECOPD) is a key area of focus when it comes to resource-saving efforts and quality improvement. ECOPD is associated with high in-hospital mortality, deterioration in quality of life, increased decline in lung function over time, and decreased expected lifetime. ECOPD is the largest component of the socioeconomic burden of living with COPD [7,8]. To reduce the burden of COPD, it is critical to make an accurate diagnosis, manage the patient effectively, and detect the patient’s symptoms early in the deterioration process [9].

The two important issues that need to be addressed are initiatives to postpone the progression of the disease. First, to maintain the patients for as long as possible in the milder stages of the disease. This will save resources and maintain the individual’s quality of life. Second, use interventions directed toward therapies that more effectively prevent or treat ECOPD. Because most exacerbations go unreported [10] and since early intervention results in better outcomes [11], the development of treatment models that enhance regular monitoring of changes in lung function over time, thereby enabling early detection of exacerbations and timely intervention [12-14].

Numerous studies have reported the use of telehealth to monitor COPD to initiate early supportive and medical interventions, supervision of medical issues, care support through reassuring conversations, telerehabilitation, and prescription renewals, among others [15-20]. Although there is a lack of consensus in the research literature regarding the effect of telehealth in the prevention of acute exacerbations, emergency room visits, and hospitalizations, as well as an increase in quality of life, telehealth services are in widespread use and considered to be effective and not to cause any harm [17,21].

The Epital Care Model (ECM) is a health- and person-centered, data-informed health service model [22], supporting the digital health service transformation informed by the World Health Organization (WHO) strategy to have integrated people-centered health services that empower individuals to be self-managed and be more active in the patient pathway [23].

Application of the ECM is mainly based on patient-reported health condition data to which the underlying service organization responds synchronously, with a range of responses including medical treatments or supervision, adapted to the patient’s reported underlying change in health or mental state.

The ECM has been tested in a number of studies during the last decade in various municipalities in Denmark [21,24,25]. One study demonstrated that using the Epital framework resulted in an increased patient understanding of their COPD, increased self-management of their condition, and a reduction of their emotional distress [24]. Another study reported that 69.4% of the severe exacerbations can be managed web-based in the patient’s own home [21]. These findings have been confirmed in an observational study in a Danish municipality (The PreCare study), which also built upon the ECM framework [25]. These studies used a combination of observational methods, surveys, and interviews.

To more robustly examine the ECM service model, we report here on a modified stepped wedge randomized controlled trial (RCT) in a group of patients with COPD. The study compares the outcomes in a group of patients with COPD receiving an ECM-based complementary telehealth service (ECTHS) compared to a group receiving usual care provided by their general practitioner (GP) and other publicly available health services.

The study had two objectives. First, to compare the mental well-being of patients with COPD and the use of COPD-related health care services being primary outcomes, and second, to compare social activities and mobility, that is, participation in cultural events and travel activities abroad, being a secondary outcome.

## Methods

### Sampling

The TEMOKAP (Danish: Telemonitorering af KOL-patienter i Almen Praksis; in English: Telemonitoring of COPD Patients in General Practice) study examined the impact of making health care more accessible through technology using a nonblinded, pragmatic, modified, stepped wedge, RCT design [26,27]. The stepped wedge consisted of blocks of 10, where those meeting on a given day at a given general practice were randomized within this block. Recruitment at each general practice was based on the availability of potential participants at a given time and alternated in the recruitment period between four general practices geographically spread in Denmark (Kalundborg and Vordingborg in the region Zealand, and Aalborg and Brovst in the region North Jutland). All practices were affiliated with a larger organization of private clinics, “allesLægehus,” with a total of 17 clinics [28]. Randomization was made within each block but not between blocks. This particular design was used as the logistics and resources available for recruiting the number of patients called for a step-by-step inclusion across the geographical locations, and also to reduce bias caused by seasonal variations. The design and research protocol did not aim to examine differences between the four general practices or to evaluate seasonal differences.

### Participants—Eligibility Criteria

Inclusion criteria were aged 45 years or older, diagnosed with COPD according to the criteria of the GOLD (Global Initiative for Chronic Obstructive Lung Disease) guidelines, have a score of minimum 3 of 6 points in cognitive screening test (The Clock Test + Three-Word Recall Memory Test) [29,30], have the ability to provide oral or written informed consent, have access to the internet in their own home, own a smartphone, and be able to use simple functions in web browsers.

Participants were excluded if they had unstable heart disease; poorly regulated diabetes; or were diagnosed with psychiatric conditions that led to mental impaired functions, such as psychotic episodes, or if they were unable to communicate in oral and written Danish.

### Method of Approach

A primary care nurse from each clinic sent out trial information and an invitation to participate in the study in the period from September 2020 to June 2021. The participants were selected from the clinic’s electronic patient record system or based on the nurses’ knowledge of potential candidates who might be suitable. Appointments were made with all interested patients to attend the clinic for an interview and a clinical examination with the principal investigator (PI).

The PI confirmed the diagnosis of COPD according to the GOLD guidelines [31]. They were classified according to their air flow limitation severity (spirometry grade 1 to 4, also named GOLD 1-4) and their GOLD risk score (GOLD ABCD Assessment Tool), assessed by the number of moderate or severe exacerbation history per year and a simple measure of breathlessness using the Modified Medical Research Council questionnaire [32,33]. The spirometric classification (GOLD 1-4) provides information regarding the severity of the patients’ airway limitation, while GOLD risk score Group A-D provides information regarding symptom burden and the risk of exacerbation, useful to guide therapy and treatment [31]. All consenting participants meeting the eligibility criteria were enrolled in the trial after reading the patient information and signing the informed consent form. All patients in both the ECTHS and the usual care group were physically examined for the purposes of the study. In addition, to ensure homogeneity of the patients in the two groups, medication adjustments were made according to patients’ GOLD groups A to D [31] to ensure that all patients were correctly medicated at the onset of the study.

### Randomization

#### Overview

After written consent, 184 patients were randomly assigned by the PI to either ECTHS or usual care using a computer-generated list of random numbers [34] for each block. The PI was not blinded in the process.

Patients were randomized in a 1:1 ratio, with a fixed block size of up to 10. The size of 10 was chosen to accommodate available clinical assessment and room capacity at each location. A total of 23 randomization blocks were conducted, with 5-10 patients randomized per block across different study visits. The variation in patient numbers per block was attributed to patient cancellations and nonattendance.

#### The ECTHS Group

**Figure 1:**
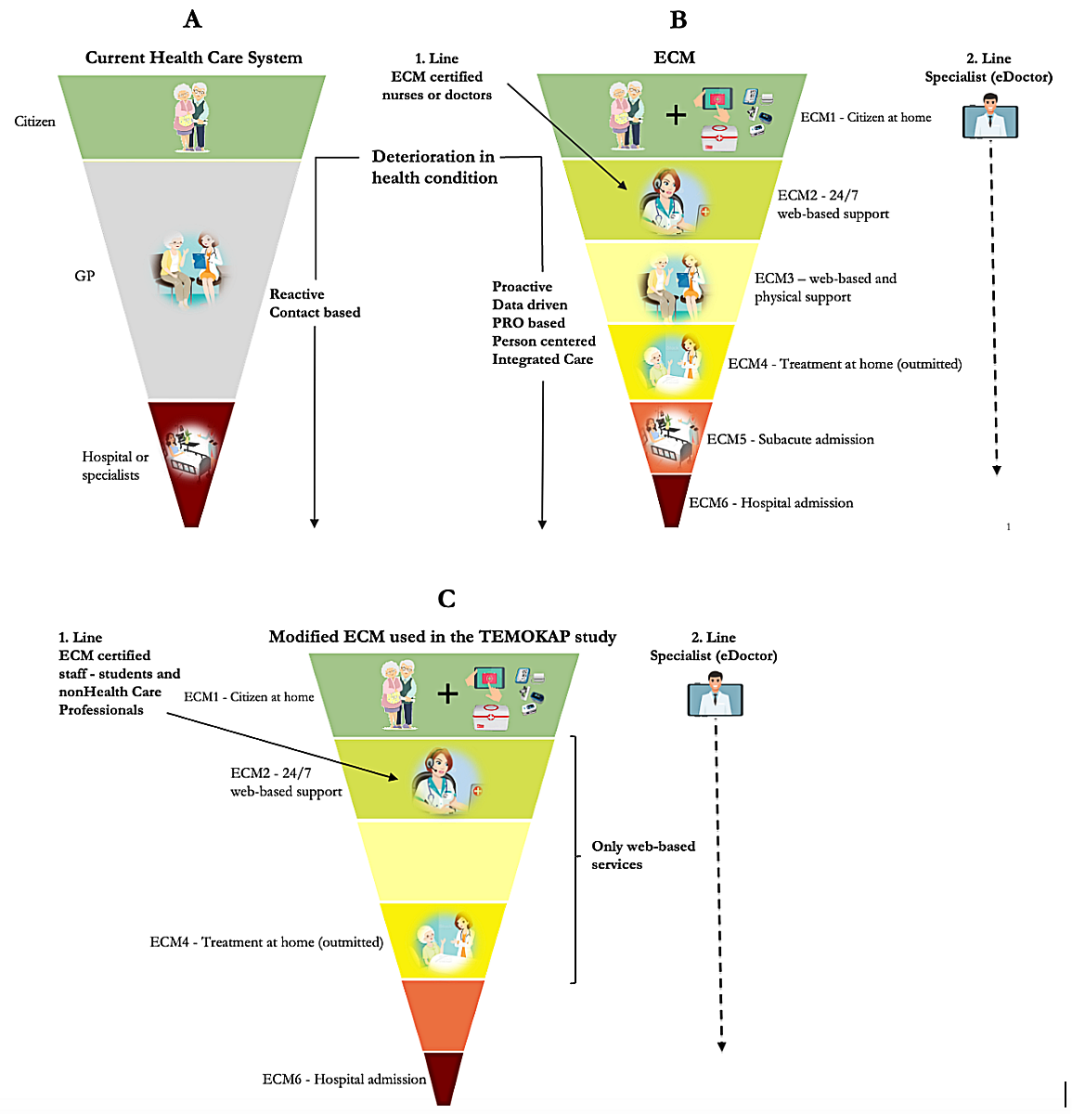
Principles and elements of the Epital Care Model (ECM) compared to a conventional health care system. (A) The conventional health care system. (B) The ECM with all service elements. (C) The modified ECM used in the TEMOKAP study. Upon medical deterioration, a wide spectrum of health services is activated and organized in the ECM guided by the incoming patient-reported outcome (PRO) data from the patient, and care activities are taken up by front-line staff. All services in the ECM are designed to mitigate the patient’s return to ECM1. Via the established telehealth solutions deployed within patients’ residences in the ECM, wherein patients can routinely report their health status, proactive intervention becomes feasible, allowing for the mitigation of potential exacerbations before they escalate into severe conditions. This stands in contrast to the conventional health care system, which is traditionally more reactive and thus has a longer latency period before the deterioration is recognized and treated. In the TEMOKAP study, a modified version (C) of the ECM was used, which consisted entirely of the web-based elements of the ECM. Thus, there was no opportunity to establish physical contact with the patients from the intervention group, who therefore only received virtual services and help through the telehealth set-up. GP: general practitioner.

The group receiving ECTHS was connected to the ECM response and coordination center (RCC), which provided the participants with 24/7/365 access to assistance from certified RCC staff who were supported by eDoctors (further details in Multimedia Appendix 1). The intervention involved the service and treatment setup from ECM 1-2 and the web-based component of ECM 4 (Figure 1). The participants were encouraged to perform their self-monitoring activities daily (saturation, pulse, lung function, temperature, and report on increased sputum, coughing, and shortness of breath). They were informed about how they should interact with the RCC. In case of deterioration indicated by the monitored data, the RCC would contact them, and they would also be welcome to initiate the contact themselves if the measurements worried them. In case of concerns or need for clarification in relation to their condition, they could contact the RCC 24/7, no matter what their measurements showed. In all cases, the RCC staff, together with the participant, would make informed decisions, via phone or video call, on how to best manage the change in condition, guided by the previous measurements evaluated with graphs, including plotted trends. Where there was a need for medical treatment, a treatment plan was drawn up with fixed follow-ups and a course plan (ECM 4). The self-monitoring activity was checked daily by the RCC, and if participants failed to return measurements for 7 days, it resulted in a call by the RCC to remedy any technical problems and encourage future regular reporting of measurements.

The participants were also equipped with an acute medicine box at home to be used when exacerbations occurred to avoid delays in the initiation of medical treatment. The content was prescribed by the eDoctor, provided directly to the participant by a local pharmacy, and used only in agreement with the RCC staff. Phanareth et al [21,22] provide further details.

#### The Usual Care Group

The group receiving usual care was provided with the usual services by their GP, emergency services from the hospital, outpatient clinics, and on-call doctors.

### Study Outcome and Measurements

#### Primary Outcome Measures

The primary planned outcome was the patient’s mental well-being as assessed by the WHO-5 Well-Being Index. This is a validated questionnaire that examines patients’ mental well-being and specifically provides measures of anxiety, stress, and depression. The Well-Being Index was measured at T0, T1, and T2 in both groups [35].

A second primary outcome was COPD-related use of health care services. These included hospital admissions, contact with GP, out-of-office services, and outpatient clinics at T0, T1, and T2 in both groups. Baseline information on hospital admissions was also examined retrospectively, 12 months from the date of inclusion. Data were self-reported and verified by the clinical coinvestigators with interviews to ensure that the COPD-related outcomes were accurate.

#### Secondary Outcome Measures

The social activities and mobility of the participants were assessed as participation in cultural events and travel abroad, respectively, at T1 and T2 by interview.

In accordance with the protocol, a third primary outcome, the participants’ technology readiness, was assessed using the Readiness and Enablement Index for Health Technology (READHY) instrument [36] at T0, T1, and T2, data to be reported elsewhere (Personal communication Palshof M 2025).

#### Baseline Data

At baseline, all demographic and clinical data were collected at the physical examination. All participants were seen by a certified health IT student (RCC staff) who assisted and supervised the patients while they completed the planned questionnaires: WHO-5 Well-Being Index [35], registration of patients’ social activities for the presented study, and the READHY instrument [36]. The RCC staff also provided the participants receiving ECTHS with the tablet and monitoring equipment and took them through a standardized training program. To ensure a high level of compliance, subsequent web-based follow-ups with the participant occurred after 1 week, 2 weeks, and 1 month. At 1 month, all participants had a conversation with the PI.

#### Follow-Up Visits

Follow-up assessments occurred at 8 and 12 months (T1 and T2) by telephone calls. Participants were interviewed by and completed all questionnaires together with the RCC staff. The staff checked the data in the electronic health record, where the patient’s contact pattern in relation to health care services is documented. In cases where there was a discrepancy between the patient’s information during the interview and the information in the RCC, the electronic health record data prevailed. As a further quality control, all the patients who had hospitalizations were contacted by a coinvestigator to ensure that the hospitalizations were COPD-related.

### Statistics

#### Sample Size Calculations

The number of required participants was calculated using “power.t.test” in RStudio (Posit PBC) using the “Two-sample *t* test power calculation” option and a fixed analytical power of 0.8 and significance level of .05 on the WHO Well-Being Index. The data used to determine the SD were taken from a group of 67 patients with COPD with GOLD risk scores B, C, or D, who were part of a pilot project that was a precursor to the TEMOKAP study. In the pilot project, the mean value was calculated to be 61.79 (SD 20.59).

Previous studies have estimated that a difference of 10 points in the WHO-5 Well-Being Index is clinically relevant [37]. The 2-sided 2-sample *t* test power calculation resulted in a sample size of 134.68 in each group. The sample was increased by 10% to ensure analytical power to accommodate an additional explanatory variable in the analysis, as well as a further 20% for potential dropouts during the study. The adjusted target sample size was therefore 194.

#### Data Analysis

The baseline data included demographic data (sex and age), FEV_1_ %pred., GOLD airflow limitation severity (1 to 4) and risk score classifications (A to D), number of comorbidities, BMI, smoking status (smoking or nonsmoking, or never smoked), “number of packages smoked per year,” MRC dyspnea score [38], admission within the last year, and WHO-5 Well-Being Index.

We report data for the WHO-5 Well-Being Index and for COPD-related contact with GPs, out-of-office services, and out-patient clinics and hospital admissions, as well as participation in cultural events and travel abroad.

We conducted two separate analyses: an intention-to-treat (ITT) analysis and a per-protocol analysis. For the analysis of the differences between the groups receiving ECTHS or usual care, in COPD-related use of health care services and participation in cultural events and travels abroad, we calculated a rate ratio with corresponding 95% CIs using Poisson regression models. Two models were tested for each variable: Model 1 was unadjusted; Model 2 was adjusted for age, GOLD risk score, time at risk, and comorbidities. The time of risk is the observation period reduced by hospitalized days.

For the analysis of differences between the ECTHS group and the usual care group on the WHO-5 Well-Being Index, we performed a 2-sample *t* test of differences between the groups and a 1-sample *t* test comparing T0 versus T1 and T2, respectively, within the groups. For all tests, a significance level of .05 was used. The open-source statistical program R (version 1.4.1717; R Core Team) was used for the analysis. The statisticians were not blinded to whether the participants were allocated to either ECTHS or the usual care.

### Ethical Considerations

The project was conducted in accordance with the Helsinki Declaration. A signed consent form was obtained from all participants after information about the project was given orally and in writing. Participants were informed that participation was voluntary and that they could withdraw from the study at any time (Multimedia Appendices 2 and 3). There was no financial compensation for participation. All the telemedicine monitoring equipment in the study was in general use and CE-marked. According to Danish practice, projects that do not involve testing of technical equipment or drugs and do not involve sampling of biological material or tissue do not require approval from an ethical committee. This has been confirmed by the ethical regional committee for the capital region of Denmark (record number: F-24055647 (2025/03/28)). The study protocol was published on the internet prior to the onset of the study [39]. To meet the requirements for publication, the study has been post registered with clinicaltrials.gov (protocol NCT06988566), mirroring the initial and public available study protocol except for that the planned primary outcome also included economic calculations, which has not been possible to conduct and that two primary outcomes contacts and usage is reported as one parameter as we could not get access to national registers as initially planned. The ECTHS in this study does not differ from the usual practice of providing services by the Epital (the ECM Clinic). No harms were therefore anticipated, and during the study, no severe events related to the study were identified. The clinic is regularly monitored by the Danish Patient Safety Authority and was last accredited on April 20, 2023. All data were stored in the Epital database, which is registered with and handled in accordance with instructions from the Danish Data Protection Agency for data protection of personal and health data [40]. The handling of data was in accordance with the General Data Protection Regulation [41]. The TEMOKAP study is reported according to the CONSORT-EHEALTH (Consolidated Standards of Reporting Trials of Electronic and Mobile Health and Online Telehealth) checklist for reporting randomized trials (Multimedia Appendix 4) [42].

## Results

### Participants

**Figure 2:**
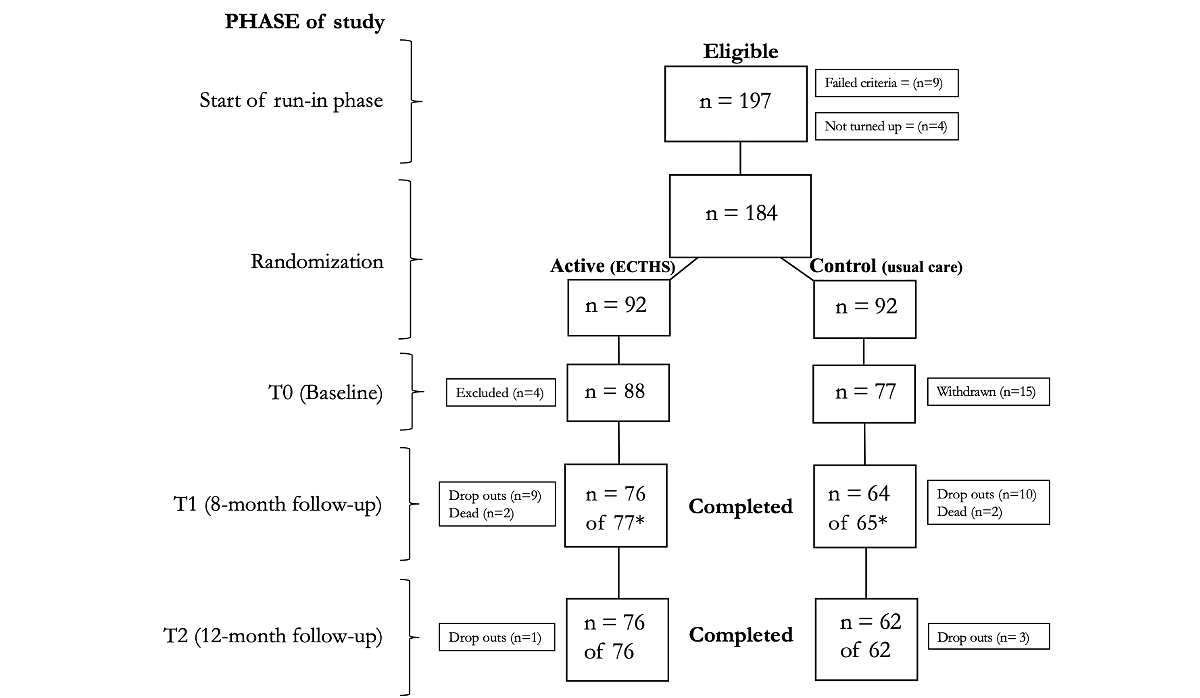
The TEMOKAP study flowchart shows the number of patients (intention-to-treat) at each phase of the study period of 12 months. T0=baseline at inclusion, T1=8-month follow-up, and T2=12-month follow-up. *Data at T1 are missing from two participants, one in each group.

Figure 2 shows the flow of the participants through the 12-month trial period and Table 1 shows the demographics and baseline parameters. A total of 197 participants were invited to one of the four clinics. Out of these, four participants did not attend, and 9 did not meet the inclusion criteria. A total of 15 participants randomized to the usual care group withdrew after being informed that they would not receive the ECTHS. In total, 4 participants were excluded after randomization, 3 due to lack of access to Wi-Fi at home and 1 due to cognitive impairment. This resulted in 165 participants at baseline, with 88 receiving ECTHS and 77 receiving usual care. At T1, the total numbers were reduced to 142 participants (76 and 66, respectively) due to withdrawals, mortality, and missing data. At T2, 4 further participants withdrew from the study, leaving 138 participants (76 and 62, respectively). One participant died before completing the telephone interview.

**Table 1:** Demographics and baseline parameters.

			ECTHS^a^ (n=92)		Usual care (n=92)		
**Age (years)**							
	Mean (SD)		67.96 (9.33)		69.39 (10.11)		
	Range, n (%)						
		<50		3 (3)		3 (3)	
		50-59		12 (13)		13 (15)	
		60-69		32 (35)		29 (32)	
		70-79		38 (41)		35 (38)	
		80-89		7 (8)		11 (12)	
		>90		0 (0)		1 (1)	
**FEV_1_% predicted^b^**							
	Mean (SD)		54.37 (18.31)		52.30(18.35)		
	Range, n (%)						
		<30		8 (9)		8 (9)	
		30-49		29 (32)		37 (40)	
		50-79		50 (54)		40 (43)	
		>80		5 (5)		7 (8)	
Co and multimorbidity, mean (SD)			1.68 (1.22)		1.75 (1.31)		
**BMI**							
	Mean (SD)		27.40 (5.82)		26.50 (6.08)		
	Range, n (%)						
		<18.5		4 (4)		2 (2)	
		18.5-24.9		30 (33)		43 (47)	
		25-29.9		27 (29)		25 (27)	
		>30		31 (34)		22 (24)	
Pack-year^c^, mean (SD)			40.40 (17.79)		38.27(16.90)		
**mMRC^d^**							
	Mean (SD)		1.78 (0.99)		1.75 (0.97)		
	Score, n (%)						
		0		1 (1)		4 (4)	
		1		46 (50)		40 (43)	
		2		25 (27)		28 (30)	
		3		12 (13)		15 (16)	
		4		8 (9)		5 (5)	
Admissions (one year prior), mean (SD)			0.26 (0.72)		0.22 (0.46)		
WHO Well-Being Index, mean (SD)			65.13 (18.55)		61.26 (21.48)		
**Sex, n (%)**							
	Female		47 (51)		43 (47)		
	Male		45 (49)		49 (53)		
**Smoke status, n (%)**							
	Smoker		36 (39)		42 (46)		
	Nonsmoker		55 (60)		46 (50)		
	Never smoked		1 (1)		4 (4)		
**Risk score, n (%)**							
	AB		58 (63)		65 (71)		
	CD		34 (37)		27 (29)		

^a^ECTHS: Epital Care Model–based complementary telehealth service.

^b^FEV_1_ % predicted: percentage of forced expiratory volume 1 second in relation to a normalized population.

^c^Number of cigarette packs smoked per year.

^d^mMRC: Modified Medical Research Council.

### WHO-5

Results from the ITT are presented below. Per protocol analyses are provided in Multimedia Appendix 5.

No differences were found between the two groups in the ITT analysis at T1 or T2 on the WHO-5 Index, where the WHO-5 score was significantly higher for the group receiving ECTHS compared to the group receiving usual care in the per-protocol analysis. This difference consisted partly of a slight increase in the group receiving ECTHS and a decrease in the group receiving usual care compared to T0. Further, no changes were found from T0 to T1 or T2 for either of the two groups in either of the analyses (Table 2).

**Table 2:** Independent 2-sample *t* test at T1 and T2 comparing the two groups and a dependent 2-sample *t* test comparing T1 and T2 to T0 on the WHO-5^a^ Index.

	ECTHS^b^, mean (SD)	Usual care, mean (SD)	*P* value (I vs C)	Estimate (95% CI)
Baseline	65.13 (18.55)^c^	61.26 (21.48)^c^	.19	–3.87 (–9.71 to 1.97)
Follow-up (T1)^d^	63.94 (21.00)^e^	61.93 (21.65)^f^	.97	0.15 (–7.42 to 7.71)
Follow-up (T2)^g^	68.76 (17.29)^e^	59.70 (19.51)^h^	.22	–4.12 (–10.75 to 2.51)

^a^WHO-5: World Health Organization-5 Well-Being Index.

^b^ECTHS: Epital Care Model–based complementary telehealth service.

^c^n=92.

^d^*P* value T1 versus T0, ECTHS: .86; usual care: .93.

^e^n=68.

^f^n=60.

^g^*P* value T2 versus T0, ECTHS: .10; usual care: .84.

^h^n=53.

### COPD-Related Usage of Health Care Services

Results from the ITT are presented below. Per protocol analyses are provided in Multimedia Appendix 5.

Table 3 shows the unadjusted and adjusted differences between the ECTHS and usual care groups in the use of health care services at both T1 and T2.

At T1, there was a reduction in COPD-related admissions of 65%, 70% in contacts to out-of-office services, and 79% in GP visits. At T2, there was a reduction in COPD-related admissions of 56%, 73% in contacts with out-of-office services, 60% in visits to outpatient clinics, and 78% in GP visits. There was a nonsignificant reduction of 49% in contacts to out-of-office service at T1. After adjustment for age, GOLD, risk time, and comorbidities, out-of-office service at T2 was not significant in the ITT analysis (Table 3). The findings in the per-protocol analysis were similar to the ITT analysis, except for a lack of significant reduction of admissions at T2 after adjustment.

**Table 3:** COPD^a^-related use of health care services T1 and T2_b_.

Period			ECTHS^c^		Usual care		Estimate (95% CI)		*P* value		Adjusted estimate^d^ (95% CI)		*P* value
**Hospital admissions**													
	T1	8^e^		19^f^		0.35 (0.15-0.78)		.01		0.38 (0.16-0.85)		.02	
	T2	14^g^		27^h^		0.44 (0.22-0.82)		.01		0.51 (0.26-0.96)		.04	
**Out-of-office service**													
	T1	5^e^		14^f^		0.30 (0.10-0.79)		.02		0.26 (0.08-0.71)		.01	
	T2	9^g^		28^h^		0.27 (0.12-0.55)		<.001		0.49 (0.21-1.07)		.09	
**Outpatient clinics**													
	T1	12^e^		20^f^		0.51 (0.24-1.02)		.06		0.59 (0.27-1.21)		.16	
	T2	18^g^		38^h^		0.40 (0.22-0.69)		.001		0.49 (0.27-0.87)		.02	
**General practitioner**													
	T1	30^e^		120^f^		0.21 (0.14-0.31)		<.001		0.24 (0.16-0.37)		<.001	
	T2	44^g^		172^h^		0.22 (0.15-0.30)		<.001		0.25 (0.17-0.35)		<.001	

^a^COPD: chronic obstructive pulmonary disease.

^b^This table shows the outcome reduction in COPD-related hospital admissions, out-of-office services, visits to outpatient clinics, and GP visits in the group receiving ECTHS compared to the group receiving usual care at T1 and T2. The outcome reduction was significant (*P*<.05) on all parameters in the group receiving ECTHS except for the outpatient clinic visit at T1 and the out-of-office services at T2. A Poisson Regression was used to calculate the estimate, CI, and *P* values. The estimate indicates the likelihood of an event occurring for those receiving the ECTHS.

^c^ECTHS: Epital Care Model–based complementary telehealth service.

^d^Adjusted for age, GOLD, risk time, and comorbidities.

^e^n=76.

^f^n=64.

^g^n=77.

^h^n=65.

### Cultural Events and Travel Abroad

Table 4 shows differences in the participation in cultural events and travel abroad between the groups receiving ECTHS versus usual care. In both the unadjusted and adjusted analyses at both T1 and T2, the group receiving ECTHS participated significantly in more cultural events and traveled abroad in comparison to the group receiving usual care. The per-protocol analysis demonstrated the same differences as in the ITT analysis.

**Table 4:** Cultural events and travels at T1 and T2^a^.

Period		ECTHS^b^	Usual care	Estimate (95% CI)	*P* value	Estimate adjusted^c^ (95% CI)	*P* value
**Cultural events**							
	T1	169^d^	49^e^	2.90 (2.13-4.03)	<.001	2.84 (2.07-3.97)	<.001
	T2	296^f^	148^g^	1.69 (1.39-2.06)	<.001	1.73 (1.42-2.13)	<.001
**Travel abroad**							
	T1	37^d^	7^e^	4.45 (2.11-10.90)	<.001	3.82 (1.79-9.44)	.001
	T2	52^f^	15^g^	2.93 (1.69-5.38)	<.001	2.50 (1.43-4.65)	.002

^a^This table shows the number and the differences in cultural events (participation in entertainment and cultural events outside of home) and travels abroad (outside Denmark) in the periods T0-T1 and T1-T2. A Poisson Regression was used to calculate the estimate, CI, and *P* values. The estimate indicates the likelihood of an event occurring for those receiving the ECTHS.

^b^ECTHS: Epital Care Model–based complementary telehealth service.

^c^Adjusted for age, GOLD risk score, risk time, and comorbidities.

^d^n=76.

^e^n=64.

^f^n=77.

^g^n=65.

## Discussion

### Principal Findings

While the ECTHS did not seem to influence participants’ self-reported well-being, it affected another primary outcome, with a reduction in COPD-related use of health care. In addition, the secondary outcome, social activity and mobility, increased in the group receiving ECTHS.

We found significant reductions in the group receiving ECTHS in almost all four measures of COPD-related use of health care services. Hospital admissions, visits to GPs, on-call doctors, emergency room visits, and outpatient attendances were all significantly lower. The data support that health services can, when supported by technology, be efficient even when manned with trained and certified nonauthorized staff. Importantly, the study demonstrates that this approach can significantly reduce health care demand and costs.

Another important finding is that the group receiving ECTHS resulted in a higher participation in cultural events and travels abroad compared to the group receiving usual care. This increase reflects the liberating effect that technology can provide individuals with COPD to increase their mobility to engage in outside activities and travel abroad.

No increase in well-being was found in the ITT analysis over time in the group receiving ECTHS or when compared to the group receiving usual care. This may reflect the limitations of general measures of well-being capability to capture the specific changes that technology brings about for individuals with COPD. In addition, the possibility of a type 2 error caused by the relatively high withdrawal of participants in the usual care group after randomization, which resulted in a lower number than calculated at T1 and T2, to be necessary to avoid a type 2 error and be able to detect a real difference between the two groups. Based on the same study data, we have performed another analysis with the inclusion of the participants who, after randomization to receive usual care, withdrew and thereafter received the same services as the intervention group outside the protocol. Here, we found an increase in well-being measured with the WHO-5 Well-Being Index (Personal Communication, Palshoff M, 2025). In addition, supporting this notion is that in the per-protocol analysis, a significant difference was found between the two groups, with a small increase over time in the group receiving ECTHS and a small decline in the group receiving usual care, resulting in a difference between the two groups at T2. In future studies, it should be considered to use mixed methods to evaluate the impact on well-being, such as interviews, either individually or as focus groups, to understand the dynamics in the participants. Such a study investigating preferences for in-person versus web-based activities in a group of participants in the TEMOKAP study found that the services based on the ECM model, including the easily accessible contact to the RCC and eDoctor, contribute to perceived well-being [43].

### Comparison to Prior Work

Our findings are in alignment with several other observational Danish studies using equivalent technology [21,24,25]. In the Danish PreCare study (PPC), the number of acute COPD-related contacts fell by 41% after inclusion in the study for at least 1 year, and in a subgroup of patients with severe COPD, costs fell by 87% (approximately US $21,186 before inclusion to approximately US $2860 after inclusion) [25]. Similar findings were obtained in another observational study by Phanareth et al [21] from 2021 with 87 patients with COPD (mean observation time 368.8, SD 248.7 days) using the same components of the ECM as in this study. Here, it was found that 69% of severe exacerbations were reversed in ECM2 without any in-person contact or use of conventional health services. These reductions in use of conventional health services in these and this study may partly be due to the 24/7 accessible RCC providing proactive, timely responses. The immediate contact in the event of deterioration not only facilitates a timely interventional response to potential issues but also serves as an educational opportunity to instruct participants on the recognition and management of changes in their condition outside of conventional health care access.

The immediate contact in case of deterioration provides not only a timely response to possible but also an opportunity to educate the participants in how to recognize and respond to changes in their condition without accessing conventional health care.

Unlike most other telehealth services, which have registered nurses or other authorized health professionals as their initial contact, in this study, the initial contact was to health education students trained and certified in the basics of COPD, and also to communicate with the participants. The use of trained undergraduate students from health education taking responsibility for patient care has been assessed in other studies. In a systematic review by Schutte et al [44] from 2015, a total of 42 articles were reviewed on student outcomes of participating in student-run clinics (SRC). The quality of care provided by students appeared adequate and was comparable with that of regular care [44]. In a recent systematic review, student learning outcomes associated with participation in SRCs were investigated. It was concluded that participation in SRCs provided students with the opportunity to develop clinical skills, foster leadership, and cultivate empathy [45]. This study contributes further evidence for this approach, and it should be considered as a tool to solve the challenges of the health care system with a shrinking workforce and a lack of resources.

The role of telehealth was an important aspect of the study reported here and is integral to the ECM. The findings reported here are supported by other studies [15,17-20,46]. A recent Cochrane review of 26 RCTs on integrated disease management (IDM) programs for COPD compared to usual care found that different IDM components had different effects. Telemonitoring had a positive effect on quality of life and physical endurance, while self-management had the greatest effect on COPD exacerbations. The authors concluded that an IDM program with a combination of exercise training, self-management, telemonitoring, and personalized education implemented in the right context should result in the best outcomes [47]. In a study by Casas et al [48] from 2006, a total of 155 patients with COPD were recruited after hospital discharge and randomized to either a standardized IDM intervention with the support of information technology or to usual care. The number of readmissions during the follow-up showed a significant decrease in admission rate in the group receiving a standardized IDM intervention with the support of information technology compared to the usual care group [48].

A recent RCT demonstrated the value of 24-hour availability for patients to contact or request a home visit from a cross-sectorial lung team. The results of a 1-year affiliation to a cross-sectorial lung team compared with a control group showed a statistically significant decrease in both the number of hospitalizations due to exacerbations as well as the length of hospital stay in the intervention group. [49]. Easy access to health care is another aspect of this study and the ECM.

### Strengths and Limitations

A strength of this study was the standardized procedure for recruitment, which included an initial examination and the standardization of medication appropriate to the patients’ COPD severity in all participants at baseline. Failure to evaluate patients at the outset and optimize medication prior to randomization is a potential confounding factor in studies of COPD.

The modified stepped wedge approach ensured even recruitment over the seasons, thereby avoiding seasonal-related deteriorations as a confounder. It should be noted that the number of deaths occurring in both the groups receiving ECTHS and usual care is low. This may be due to the participants being recruited from general practices, and in particular, the number of people in GOLD severity group 4 may be lower than reported in other studies.

The study has several limitations. One is that the use of COPD-related health care resources and social activities was self-reported and only gathered at T1 and T2, and may introduce some recall errors. To limit this bias, one of the authors (LS) contacted all participants by telephone and rigorously examined the history of each participant with respect to hospitalization. The original plan was to extract patient data from national systems, but this turned out not to be feasible. If we had been aware of this problem prior to the study, we could have requested participants to complete a diary of their activities over the period of the study. As data were sampled at two different timepoints based on participants’ recall of events over a longer period, we considered each of these timepoints to be separate observations. It can be argued that it is a longitudinal study and that the generalized estimating equations analysis would be a more appropriate method. This should be considered in future studies, including the design to ensure that the data can be handled as a longitudinal sample. Another statistical-based limitation is that the study was not blinded to either the PI, who randomized the participants, or to the researchers who performed the analysis. In addition, due to the nature of the study, only those in the ECTHS group were in regular contact with the RCC and thereby known to the staff who five of the coauthors belonged to. All participants were to a certain extent still receiving usual care from the GP clinics and other health care services, which were not always aware of whether the participants also received ECTHS. This may have caused a risk of bias. One example is how a number of participants allocated to usual care retracted from the study when being told that they would not receive ECTHS. In addition, there may be a risk that the investigators unconsciously favor certain decisions to achieve more beneficial data. The authors have been aware of this challenge, which is common in telehealth interventions, and have made every effort to be transparent and work rigorously to be as neutral as possible. A further limitation is that the study is confined to a special setting of GPs’ clinics, which are organized in a particular way. To determine the generalizability of these findings, further studies spanning diverse regions and organizations are required. This is due to the inherent variability in how COPD patient management and care are organized across different health care environments, which serve communities with characteristics that may differ from the population studied here. [50].

### Perspectives

The study contributes two important findings to the work on the management of COPD. First, a primarily web-based service can reduce contacts with other health services and associated costs, along with providing a feeling of confidence in participants to increase their social activities. Second, the frontline staff can consist of trained and certified individuals without extensive education in health care, thereby reducing the burden of staff in health care services, along with a further reduction in costs.

These findings should be confirmed in larger-scale studies and more examined in populations of older adults with other LTHC.

### Conclusions

Telehealth services, building on the ECM and offered as complementary to GPs’ usual services for patients with COPD, reduce the number of COPD-related health care service use and increase the social activity and mobility of participants.

The study demonstrated that health care service can be provided efficiently with a reduced burden on the regular workforce by using trained and certified nonhealth professionals in the frontline of care.

The study thereby contributes to the growing body of knowledge on the benefits of web-based health care and digital transformation in the care of people with LTHC and, in particular, COPD.

## Appendix

Multimedia Appendix 1 The Epital Care Model and the staff.

Multimedia Appendix 2 Patient information.

Multimedia Appendix 3 Consent form.

Multimedia Appendix 4 CONSORT 2010 checklist.

Multimedia Appendix 5 Per protocol analysis.

## Abbreviations

CONSORT-EHEALTH: Consolidated Standards of Reporting Trials of Electronic and Mobile Health and Online Telehealth
COPD: chronic obstructive pulmonary disease
ECM: Epital Care Model
ECOPD: chronic obstructive pulmonary disease exacerbations
ECTHS: Epital Care Model–based Complementary Telehealth Service
GP: general practitioner
GOLD: Global Initiative for Chronic Obstructive Lung Disease
IDM: integrated disease management
ITT: intention-to-treat
LTHC: long-term health condition
PI: principal investigator
RCC: response and coordination center
RCT: randomized controlled trial
READHY: Readiness and Enablement Index for Health Technology
SRC: student-run clinics
WHO: World Health Organization

## Editorial Notice

Due to external challenges during the planning phase, including the COVID-19 pandemic, the TEMOKAP study was inadvertently not registered in a public trial registry in a timely manner. However, we assess that this oversight did not affect the trial’s conduct or results, which are in full accordance with the original protocol. The trial registration number has now been included in the abstract with the addendum “(retrospectively registered).”

## Data Availability

The datasets generated or analyzed during this study are not publicly available because they are considered personal health data, and sharing is not consented to in the consent form. As the authors support the FAIR principles, they can be contacted to discuss the sharing of subsets in an anonymous form.
