# Supplementary material for: Reduced Chronic Obstructive Pulmonary Disease–Related Utilization of Health Care Services and Increased Social Activities by Patients Offered a 24/7 Accessible Telehealth Service Based on the Epital Care Model: Pragmatic Modified Stepped Wedge Randomized Controlled Trial
Source: J Med Internet Res. 2025 Oct 22;27:e65300. doi: 10.2196/65300 (PMC12590041; doi:10.2196/65300)
Supplement: Multimedia Appendix 1 [file jmir_v27i1e65300_app1.docx]

The Epital Care Model and the staff

The overall objective of the ECM is to ensure that patients, to the greatest extent possible and for the longest possible time, are in the state of "ECM 1", the first stage of the ECM model characterized by; the greatest possible freedom, independence, and self-control (active and independent living), regardless of whether at home or outside the home (see Fig 1). Even for patients with severe chronic disease, ECM 1 represents the level that the ECM service will explicitly try to keep the patient in, or attempt to return the patient to. This provides the state of greatest independence for the patients. In addition the patients have the security of knowing that healthcare services are available 24/7 should a need for help arise. Another objective of the ECM is to be able to delegate as much responsibility and control to the patient as possible. This is supported by empowerment-promoting measures such as structured conversations and the provision of enabling technologies which may help the patient monitor themselves under continuous supervision. The patient is encouraged to act for themselves and respond appropriately to changes in conditions such that the classic passive patient role is reduced as much as possible. If their condition worsens, the patients are supported virtually in ECM 2 by specially trained staff from a Response and Coordination Centre (RCC). All patients in ECM are equipped with "emergency medicine" (broad-spectrum antibiotics plus oral prednisolone), which is kept by the patient at home, and which is only used in consultation with the ECM staff. If the deterioration requires urgent medical treatment, the eDoctor is involved, and a plan is drawn up for a treatment course, that is carried out by the patient under supervision and monitoring by the RCC staff. In rare cases in which virtual support is not sufficient, a physical visit to the patient’s home from health care professional will be arranged to clarify the situation in the context of ECM 3-6 ^22^.

This study is focussed on examining the effects of the virtual part of ECM. This therefore involved the organizational service and treatment setup from ECM 1 - 2 and the virtual part of ECM 4 that was used in the group receiving ECTHS (se fig.1).

**The eDoctor**

The eDoctor is primarily responsible for patient care within the ECM Clinic. This responsibility is delegated to specific areas of the clinic's RCC staff through detailed instructions and delegated authority.

The eDoctor conducts pre-qualification of individual COPD patients in connection with the initiation of treatment at the ECM Clinic.

RCC staff work under the delegation and responsibility of the eDoctor and perform tasks related to ongoing monitoring of patient measurements, prescription medication management, initiation of exacerbation treatments, follow-ups, ensuring compliance with medication and devices, care and support conversations, technical support, and ongoing documentation.

A crucial prerequisite for consistently performing work according to current healthcare guidelines is that RCCstaff have the opportunity to consult with the eDoctor on an ongoing basis, and that the eDoctor continuously supervises the work at the ECM Clinic.

eDoctor's tasks:

- Pre-qualifies patients who wish to be affiliated with the ECM Clinic
- Conducts virtual "ward rounds"
- Is available to ECM Clinic staff 24/7
- Is responsible for treating patients affiliated with the ECM Clinic
- Is responsible for initiating medical treatments
- Is responsible for certification and supervision of the ECM Clinic's RCC staff
- Is available to the patient when relevant.

**The RCC-staff in the ECM Clinic**

RCC staff are employed by the ECM Clinic and work under the instructional authority of the ECM Clinic's managing physician, who may also act as an eDoctor.

Contact with telemedicine patients is based on telephone or video contact, in combination with the patient's own condition measurements. The RCC staff conducts a thorough and precise assessment of the patient's condition based on the patient's measurements and status. The combination of RCC staff's personal contact with the individual patient in connection with inclusion and TM certification for the ECM Clinic, telemedicine contact in connection with changes in the patient's condition, and background information about the patient in the ECM Clinic's journal system enables RCC staff to work holistically in their approach to patients affiliated with the ECM Clinic. This also makes it possible to assess which interventions best serve the individual patient in the specific situation.

This means that RCC staff in the ECM Clinic handle a combination of nursing tasks of an instrumental clinical nature and simultaneously assess needs that cover the patient's entire health and social situation.

In other words, RCC staff in their work with early detection, timely intervention, and treatment management, including complex and unforeseen patient cases, draw on knowledge and skills that extend beyond acute nursing care and contribute to ensuring quality, consistency, and patient safety in the ECM Clinic.
RCC staff work, as mentioned, on delegation and with the instructions and job descriptions that cover the healthcare tasks performed under the auspices of the ECM Clinic's various functions.

The frontline staff at the RCC were not trained physicians or registered nurses but students from health-related programs at the faculty of health and medicine at the University of Copenhagen and Danish Technical University. All staff were certified with both a practical and theoretical test after four weeks of hands-on training in the RCC function. RCC's frontline staff worked under the authority of the eDoctor, who was available 24/7/365 and in all contexts the person responsible for the treatment of the patients in the group receiving ECTHS. Further details on the system and the organization have been reported earlier elsewhere ^21,22^.

**Certification of the RCC staff**

A prerequisite for being an RCC staff member in the ECM Clinic is to be a student or graduate in one of the healthcare programs (e.g., health IT or nursing) at a University of Applied Sciences or a University, and to have completed ECM certification.

ECM certification consists of a hands-on training lasting between 5-7 days, as well as a theoretical and practical exam. The certification takes place in collaboration with examiners from the University of Copenhagen, ensuring a uniform and adequate level of competence.

**Curriculum – ECM Certification**

All written materials and instructions for the following curriculum are collected in the ECM Clinic's instruction folder in Dropbox (EH-instructions 2022), which only ECM Clinic employees are invited to and may access. It is assumed that ECM-certified employees understand and are familiar with all clinical, technical, and administrative instructions and have knowledge of the articles and documents in the instruction folder.

**General Healthcare**

The certified employee must be able to:

- Account for the healthcare system's most significant challenges
- Account for the healthcare system's sector division and explain the individual sectors' work areas and tasks they perform
- Explain the healthcare system's payment methods and how they affect the individual sectors
- Describe the difference between the system-centric and the person-centered healthcare system

**General about ECM**

The certified employee must be able to:

- Account for the ECM model's 6 domains (content, activities, and staffing)
- Account for the ECM model's dynamic and adaptive response based on PROM
- Explain how ECM can facilitate a paradigm shift toward a person-centered healthcare system
- Explain the ECM model's proactive elements and how patient involvement promotes empowerment
- Explain the most important elements that distinguish ECM from a conventional healthcare system

**ECM-relevant Disease Areas**

***Chronic Obstructive Pulmonary Disease (COPD)***

- General about COPD
  - Account for the causes of COPD and the clinical presentation
  - Account for the symptoms of COPD
  - Explain how the diagnosis of COPD is made
  - Account for the GOLD guideline classifications of severity levels (1,2,3,4 and A,B,C,D)
  - Describe the most important comorbidities of COPD

*Treatment of stable COPD*

- Explain the medical treatment principles for the different severity levels of COPD
- Account for the content and effect of inhalation medication SABA, SAMA, LABA, LAMA, ICS
- Account for the different types of devices for inhalation medication
- Describe the different formulations of inhalation medication (powder, spray, liquid) and account for the advantages and disadvantages of the different types
- Account for the possibilities of other medical treatment of COPD, in addition to inhalation treatment - i.e. oral prednisolone, Daxas, fixed low-dose antibiotics, home oxygen therapy, etc.
- Be able to advise and guide patients in the use of the different device types (pMDI, pMDI + spacer, DPI and nebulizer systems)
  - Be able to advise and guide patients regarding the most important side effects of the medication
  - Have knowledge of non-pharmacological treatment options for COPD
  - Know the Danish guidelines and care pathways for COPD

*Treatment of acute COPD*

- Account for the symptom picture in acute exacerbation
- Account for the principles of medical treatment of acute COPD (exa1,2,3)
- Explain the treatment process (procedures, workflows, start of treatment, monitoring, follow-up, and completion) for initiated exacerbation treatments

*COPD comorbidities*

- Describe the most important comorbidities of COPD (heart failure, diabetes, anxiety/depression, osteoporosis, cancer)
- Most important manifestations
- Overall treatment principles
- Account for how the EECM Clinic supervises in relation to comorbidities

***Asthma***

The ECM Clinic has a number of asthma patients affiliated, and it is therefore important that ECM certified employees have an overall knowledge of the disease, the clinical manifestations, and the most common treatment principles (curriculum: Read Asthma-Epitalinfo in the folder "Clinical instructions").

*General about asthma*

- Account for the causes of asthma and the clinical presentation
- Account for the symptoms of asthma
- Describe GINA guideline classifications of severity levels (1,2,3,4,5)

*Treatment of acute asthma*

- Account for the symptom picture in acute asthma exacerbation
- Account for the principles of medical treatment of acute asthma (exa1,2,3)
- Explain the treatment process (procedures, workflows, start of treatment, monitoring, follow-up, and completion) for initiated exacerbation treatments

**IT support for the RKC function**

The certified employee must have a basic understanding of the ECM Clinic's underlying two IT systems (Appinux and EH) regarding the most important functionalities. It is also expected that the certified employee can navigate both systems, understand and use the treatment applications, be familiar with the documentation systems, and be able to navigate the graphical representations of patient data to assess and act on patients' condition changes.

*Appinux – the patient-oriented part*

The certified employee must have a basic knowledge of:

- The ECM patient's monitoring equipment (tablet, spirometer, pulse oximeter, temperature meter, acute medication)
- The elements included in the ECM, EH-lite subscription
- The applications on the patient's tablet

*Appinux – the healthcare professional part*

Be familiar with all the following service catalogs in Appinux:

- Service response
- Decision basis
- Medication status
- Onboarding - members Be familiar with and able to extract the following customer-specific reports in Appinux:
- "Member control stats in date interval"
- "Patient without measurements today"

*ECM – Website and back-office platform*

The certified employee must have a basic knowledge of:

- The content of the clinic's website
- Functions and applications for the patient
- All tools and applications dedicated to "Staff":
  - Create patient
  - Alarms
  - Journal notes
  - Registrations
  - Reports
  - Mental training

**Processes and Workflows**

*The certified employee must be able to:*

- Create a new member
- Conduct an onboarding guided by the service catalog
- Guide the patient in conducting daily measurements
- Adjust medication according to severity guided by "Medication status"
- Ensure legal formalities (consent form, etc.)
- Handle acute calls from patients
- Handle red measurements
- Handle multiple and repeated yellow measurements
- Handle follow-ups
- Handle missing measurements
- Understand the indication for and initiate exa-treatments
- Plan exa-processes, follow-ups, and conclude processes
- Register and document in "Service response"

*Technical Procedures*

- Configure tablets
- Start Quick support
- Guide patients in the log-in procedure
- Be able to guide patients with technical problems with:
  - Spirometer
  - Pulse oximeter
  - Tablet
  - Internet connection via Wi-Fi

**Communication and Empowerment**

It is expected that certified employees know the elements of good communication and have trained in communication with our patients. Know the definitions and principles of empowerment and the empowerment-promoting approach in communication and services (listen to the empowerment podcast on the website). The following are guidelines for what is expected to be learned.

*The certified employee is expected to:*

- Be able to master communication at eye level
- Be service-minded and solution-oriented in all situations
- Know their limitations and understand when and how to get help and support to perform their tasks with patients
- Continuously develop the empowerment-promoting approach in their communication

**Certification**

The certification consists of a theoretical and practical exam, scheduled for 30 and 20 minutes, respectively. The practical exam will be based on a case, with the possibility that it can take place in a live session with a "real citizen" - alternatively, it will be a constructed case.

- The ECM Clinic's managing physician is responsible for the examination in collaboration with an external examiner invited to the certification
- A guiding grade is given that is for internal use only (can be informed individually if desired)
- Externally, the certification will be assessed as passed or failed
- All certified persons will be issued an ECM certification certificate
- Upon passing the certification, a document on Delegated Prescribing Right is signed (see instruction folder)

**Formalities**

- The certified person must be able to present a signed employment contract with the ECM Clinic
- The certified person must have signed EH's current confidentiality statement
- The certified person must have signed a document on delegated prescribing rights (see instruction folder)
