# Supplementary material for: Reduced Chronic Obstructive Pulmonary Disease–Related Utilization of Health Care Services and Increased Social Activities by Patients Offered a 24/7 Accessible Telehealth Service Based on the Epital Care Model: Pragmatic Modified Stepped Wedge Randomized Controlled Trial
Source: J Med Internet Res. 2025 Oct 22;27:e65300. doi: 10.2196/65300 (PMC12590041; doi:10.2196/65300)
Supplement: Multimedia Appendix 2 [file jmir_v27i1e65300_app2.docx]

**Telemonitoring and Treatment of COPD in General Practice (TEMOKAP Study)**

We would like to ask you if you would like to participate in a research project that we call the “TEMOKAP study”.

The purpose of this project is to investigate whether home-based treatment with telemedicine and support from healthcare professionals can prevent acute exacerbations of COPD and avoid acute hospital admissions, and whether this will improve the participant's health status and quality of life. In other words, the project seeks to document how life with COPD can be made better and safer for all COPD patients.

The project is a collaboration between the University of Copenhagen, alles Lægehus (everyone's medical practice), Epital Health A/S, and Apopro.dk and will be conducted as a scientific study.

**Why the TEMOKAP Study?**

The TEMOKAP study represents a new treatment method where, with the help of modern technology and telemedicine, we can offer examination and treatment in your own home if a medical issue or an acute exacerbation of your chronic illness should arise. In a time of Corona, as Denmark is experiencing, this principle is fully in line with the Danish Health Authority's recommendations to protect our chronic patients and reduce the risk of infection.

**What are the benefits of participating?**

- Direct and easy daily access to medical/healthcare professional help regarding your COPD
- The possibility of receiving treatment in the safe environment of your own home
- Free delivery of medication to your home address, so you don't have to go to the pharmacy yourself
- Reduced risk of infection with the Corona virus and other infectious diseases
- Prevention of exacerbations of your COPD and avoidance of hospital admissions
- Increased co-determination in your own treatment process
- Freedom and security to live better with a chronic illness

**How does it work?**

The TEMOKAP study works “proactively”, meaning it reacts and acts “before the damage is done”. This has become possible through the tele-tools that you will be provided with and use daily to take measurements from home. Your measurements are analyzed daily by mathematical models that can help the TEMOKAP study's doctors and nurses predict exacerbations, so they can initiate treatment much earlier than normal.

As a participant in the TEMOKAP study, in addition to your usual medication, you will have two types of “acute medication” (antibiotics and corticosteroids) readily available at home. This means that our doctors and nurses can initiate treatment immediately if an exacerbation should occur. You avoid a lot of hassle with getting prescriptions and picking up medication at the pharmacy – and most importantly, you save time and can start treatment quickly. TEMOKAP study, participant information (v3) 2

The TEMOKAP study also has a strong focus on your well-being and safety and therefore offers an open emergency line where you can get in touch with qualified healthcare professionals who are always ready if you need help or support in your daily life.

**What does participation involve?**

Initially, you will undergo a clinical examination by a doctor or nurse from the TEMOKAP study with a special focus on your COPD. We will adjust your medication if necessary, and together we will make a plan for your wishes and goals for the future. You will then decide whether you wish to participate in the TEMOKAP study, and if you meet the necessary criteria for participation, you will need to sign a “Consent Form”. Once signed, you will be randomly placed in either an “active group” or a “control group”.

**Active group**

As a participant in the “active group”, you will be provided with an electronic screen (tablet) with a direct connection to the TEMOKAP study's doctors and nurses, as well as a spirometer and an oximeter. You will be trained in how to use the equipment to take your daily measurements and learn how to contact us if you need to.

Treatment, access to doctors and nurses, all technical equipment, and the delivery of medication are completely free for you. The only thing you must pay for is your usual medication and the acute medication that you need to have readily available at home. The acute medication costs approximately DKK 50 after subsidy.

Electronic screen (tablet) Spirometer Oximeter

Your participation in the TEMOKAP study will last for one year. At the start of the project, after 6 months, and at the end of the project, we will collect a series of health information about your health, which we will analyze to investigate how you have been and to compare with a “control group”. This will take place a total of three times, through questionnaires and consultations that you will be invited to.

You will continue to be affiliated with alles LægeHus, so you can contact and visit your own doctor, just as you usually do, and if you are connected to a hospital outpatient clinic, this will also continue – as usual.

**Control group**

As a participant in the “control group”, you will continue your treatment as usual through alles LægeHus. At the start of the project, after 6 months, and at the end of the project after 1 year, you will be contacted by one of the TEMOKAP study's staff members who will collect a series of health information about your health to investigate and analyze how you have been. The results will be compared with the “Active group” to investigate the effect of the two treatments. This will take place a total of three times, through questionnaires and consultations that you will be invited to or called about.

**Applicable to both the “Active group” and the “Control group”** TEMOKAP study, participant information (v3) 3

You must give permission for all your health information relevant to conducting the study to be obtained from alles Lægehus's practice system, Sundhed.dk (Health.dk), the Shared Medication Card (FMK), national quality databases (DR-KOL, the National Patient Registry), as well as from Epital Health’s clinical database. The collected information will only be used by healthcare professionals, analysts, suppliers, and researchers who have a documented affiliation with the study. All personal information will be processed and stored in a secure database at Epital Health A/S and will be treated with full confidentiality and in accordance with the requirements of the authorities.

Information registered about you in connection with the treatment will, like other health information, be stored for 10 years after the project's completion. Data included in research results will be stored in anonymized form indefinitely.

Your participation is entirely voluntary, and if you no longer wish to participate, it will not affect your relationship with alles LægeHus, which will simply continue as usual.

You can withdraw your consent to participate at any time by contacting alles LægeHus or Epital Health, without necessarily giving a reason. This will not affect your relationship with either alles LægeHus or your relationship with the local hospital. If you do not wish to participate, your treatment will proceed as normal with your own doctor at alles LægeHus, at the hospital, or through the on-call doctors.

Participation in the TEMOKAP study does not entail any increased risk compared to the treatment normally offered by the healthcare system.

There may be circumstances that will lead to your participation being terminated and you being withdrawn from the trial. This could be, for example, if another life-threatening or disabling illness should occur during the trial period, or if the progression of your underlying disease reaches a severity that requires special measures, including intensive care and treatment at a hospital or other institution.

You have the right to object to, access, and request rectification of the records made in connection with your involvement in the TEMOKAP study. You have the right to request the deletion of information that the TEMOKAP study has registered about you.

You also have the right to complain to the Danish Data Protection Agency (Datatilsynet) if you are dissatisfied with the way the TEMOKAP study processes your personal data. The Data Protection Agency's contact information can be found at www.datatilsynet.dk.

**Project Management**

Epital Health A/S Klaus Phanareth, Chief Medical Officer, Consultant Physician, PhD, Specialist in Internal Medicine and Respiratory Medicine. Email: info@epital.com, telephone: 93 999 111

alles Lægehus Contact the TEMOKAP study by telephone 93 999 111 or email: info@epital.com
