# Supplementary material for: Reduced Chronic Obstructive Pulmonary Disease–Related Utilization of Health Care Services and Increased Social Activities by Patients Offered a 24/7 Accessible Telehealth Service Based on the Epital Care Model: Pragmatic Modified Stepped Wedge Randomized Controlled Trial
Source: J Med Internet Res. 2025 Oct 22;27:e65300. doi: 10.2196/65300 (PMC12590041; doi:10.2196/65300)
Supplement: Multimedia Appendix 3 [file jmir_v27i1e65300_app3.docx]

**Telemonitoring and Treatment of COPD in General Practice**

I hereby confirm that I have received participant information about the study regarding “telemonitoring and treatment of COPD in general practice” and understand that I will be randomly assigned to either an “active group” or a “control group”.

Furthermore, I have had the opportunity to ask questions and have received satisfactory answers.

I understand that my participation in the study is entirely voluntary and that I can withdraw my consent to participate at any time by contacting Epital Health A/S, without necessarily providing a reason. This will not affect my relationship with either my general practitioner or my local hospital.

The study is a clinical trial aimed at investigating the effect of supporting COPD patients in general practice with telemedicine-based home measurements to prevent and hinder exacerbations, thereby increasing quality of life. The project is a collaboration between the University of Copenhagen, alles Lægehus, and Epital Health A/S and will be conducted as a scientific study based on offers of participation made to relevant patients affiliated with alles Lægehus A/S.

I give permission for health information relevant to conducting the study to be obtained from alles Lægehus's practice system, Sundhed.dk (Health.dk), the Shared Medication Card (FMK), national quality databases (DR-KOL, the National Patient Registry), registers and databases, as well as from Epital Health’s clinical database. I am aware that the information will be used by healthcare professionals, analysts, and researchers who have a documented affiliation with the study, and that my information will be processed and stored in a secure database at Epital Health A/S. All data will be treated with full confidentiality and in accordance with the requirements of the authorities.

As a participant in the “active group”, I will be provided with a tablet (an electronic screen), a spirometer, and a pulse oximeter, which I will use to take my daily condition measurements. The electronic screen has exclusively had programs installed to support my measurements and to conduct telephone or video consultations. This includes sensitive personal data. The electronic screen is not locked and must therefore be stored securely. If the tablet is stolen, it is important that I immediately inform a member of the study staff so that the data can be remotely deleted. I will still maintain my contact with alles Lægehus even though I am participating in the study and can contact my own doctor at any time if I wish to.

I understand that as a participant in the “active group”, I must acquire and pay for two types of acute medication, dispensed by my pharmacy, containing prescribed medication for the rapid initiation of treatment in the event of acute exacerbations of my health condition. These costs also include refilling the medication box as needed. The price for the medication is approximately DKK 242 without subsidy and approximately DKK 50 with subsidy.

As a participant in the “control group”, I will continue my current treatment with my own doctor and follow the current control intervals. TEMOKAP study, consent form (ver.4) 2

The study “Telemonitoring and Treatment of COPD in General Practice” only assumes responsibility for the treatment performed while you are in Denmark. For travel abroad, treatment proceeds as normal through your general practitioner and via any travel insurance taken out.

Information registered about me in connection with the treatment will, like all health data, be stored for 10 years after the project's completion. Data included in research results will be stored in anonymized form indefinitely.

I have the right to object to, access, and request rectification of the records made in connection with my involvement in the study. I have the right to request the deletion of information that has been registered about me during my participation in the study.

I have the right to complain to the Danish Data Protection Agency (Datatilsynet) if I am dissatisfied with the way the study processes my personal data. The Data Protection Agency's contact information can be found at www.datatilsynet.dk.

I know enough about the purpose, method, advantages, and disadvantages of the trial to say yes to participating.

I have received a copy of this consent form.

I wish / do not wish to be informed about the research project's results and any potential consequences for me.

**To be completed by the study participant:**

____________________________, _____________, __________________________________.

Name (block letters) Date Participant's signature

**The following to be completed by the informing study staff member:**

The undersigned staff member from the study confirms that sufficient participant information, both verbal and written, has been provided to allow a decision to be made regarding participation, and that the above person has agreed to participate under the described circumstances.

A copy of this consent form has been given to the participating person.

______________, ____________________________________.

Date Signature of the informing study staff member

**Study Management**

Klaus Phanareth, Chief Medical Officer, Consultant Physician, PhD, Specialist in Internal Medicine and Respiratory Medicine. Phone: 93 999 111, email: info@epital.com

Contact the study by telephone 93 999 111 or email: info@epital.com
