# Supplementary material for: Reduced Chronic Obstructive Pulmonary Disease–Related Utilization of Health Care Services and Increased Social Activities by Patients Offered a 24/7 Accessible Telehealth Service Based on the Epital Care Model: Pragmatic Modified Stepped Wedge Randomized Controlled Trial
Source: J Med Internet Res. 2025 Oct 22;27:e65300. doi: 10.2196/65300 (PMC12590041; doi:10.2196/65300)
Supplement: Multimedia Appendix 5 [file jmir_v27i1e65300_app5.docx]

The process of determining whether the patients failed to qualify to be included in the per protocol analysis was according to the following criteria: 1) If no measurement was made within the first month,

2) If the patient indicated that they did not want to continue in the study,

3) If the patient had not treatment contact with the RCC,

4) If the home monitoring kit was returned (with or without notice).

| **Table S1:** Two-sample t test at baseline, T1 and T2 with WHO-5 as outcome variable – per protocol analysis. | | | | |
| --- | --- | --- | --- | --- |
|  | **ECTHS, Mean (SD)** | **Usual Care, mean (SD)** | **Estimate, [CI]** | **P-value** |
| **Baseline** | 65.13 (18.55)^a^ | 61.26(21.48)^a^ | -3.87 [-9.71;1.97] | .19 |
|  |  |  |  |  |
| **Follow-up 8 months** | 63.50(21.53)^b^ | 61.93(21.65)^c^ | -1.57 [-9.25;6.12] | .69 |
| **P-value T1 vs. T0** | .75 | .93 |  |  |
| **Follow-up 12 months**  **P-value T2 vs. T0** | 68.68(17.40)^d^  .11 | 59.70(19.51)^e^  .84 | -8.98 [-15.80;-2.16] | .01 |

*a: n = 92*

*b: n = 64*

*c: n = 60*

*d: n = 65*

*e: n = 53*

| **Table S2:** COPD related utilization of healthcare services 0-8 months – Per-protocol analysis | | | | | | | |
| --- | --- | --- | --- | --- | --- | --- | --- |
|  | **Period** | **ECTHS** | **Usual Care** | **Estimate, [CI]** | **P-value** | **Adjusted estimate^a^, [CI]** | **P-value** |
| **Admissions** | T1 | 8^b^ | 19^c^ | 0.38 [0.16;0.84] | .02 | 0.41 [0.17;0.91] | .04 |
|  | T2 | 14^d^ | 27^e^ | 0.47 [0.24;0.88] | .02 | 0.54 [0.27;1.02] | .07 |
|  |  |  |  |  |  |  |  |
| **Out-of-office service** | T1 | 5^b^ | 14^c^ | 0.32 [0.10;0.84] | .03 | 0.28 [0.09;0.76] | .02 |
|  | T2 | 9^d^ | 28^e^ | 0.29 [0.13;0.59] | < .01 | 0.52 [0.22;1.12] | .11 |
|  |  |  |  |  |  |  |  |
| **Outpatient clinics** | T1 | 12^b^ | 20^c^ | 0.54 [0.26;1.09] | .06 | 0.63 [0.29;1.21] | .21 |
|  | T2 | 18^d^ | 38^e^ | 0.43 [0.24;0.74] | < .01 | 0.52 [0.24;0.93] | .02 |
|  |  |  |  |  |  |  |  |
| **General Practitioner** | T1 | 29^b^ | 120^c^ | 0.22 [0.14;0.32] | < .01 | 0.25 [0.16;0.38] | < .01 |
|  | T2 | 35^d^ | 172^e^ | 0.18 [0.13;0.26] | < .01 | 0.21 [0.14;0.30] | < .01 |

^a^Adjusted for age, GOLD*, risk time, and comorbidities.

^b^n=71

^c^n=64

^d^n=72

^e^n=65

*Table S2, shows the outcome reduction in admissions, out-of-office services, visits to outpatient clinics and GP visits in the group receiving ECTHS compared to the group receiving usual care at T1 and T2. The outcome reduction was significant (p<0.05) on all parameters in the group receiving ECTHS except for the outpatient clinic visit at T1 and the out-of-office services at T2. A Poisson Regression was used to calculate the estimate, CI, and p-values. The estimate indicates the likelihood of an event occurring for those receiving the ECTHS.*

| **Table S3:** Cultural events and travels after 8-month follow up Per-Protocol Analysis | | | | | | | |
| --- | --- | --- | --- | --- | --- | --- | --- |
|  | **Period** | **ECTHS** | **Usual Care** | **Estimate, [CI]** | **P-value** | **Estimate**  **Adjusted^a^, [CI]** | **P-value** |
| **Cultural events** | T1 | 165^b^ | 49^c^ | 3.04 [2.23;4.22] | < .01 | 2.93 [2.13;4.11] | < .01 |
|  | T2 | 290^d^ | 148^e^ | 1.77 [1.45;2.16] | < .01 | 1.80 [1.48;2.21] | < .01 |
|  |  |  |  |  |  |  |  |
| **Travel abroad** | T1 | 37^b^ | 7^c^ | 4.76 [2.26;11.67] | < .01 | 4.11 [1.92;10.19] | < .01 |
|  | T2 | 52^d^ | 15^e^ | 3.13 [1.81;5.76] | < .01 | 2.66 [1.52;4.95] | < .01 |

^a^Adjusted for age, GOLD, risk time, and comorbidities.

^b^n=71

^c^n=64

^d^n=72

^e^n=65

*Table S3 shows the number and the differences in cultural events (participation in entertainment and cultural events outside of home) and travels abroad (outside Denmark) in the periods T0-T1 and T1-T2. A Poisson Regression was used to calculate the estimate, CI, and p-values. The estimate indicates the likelihood of an event occurring for those receiving the intervention.*
